# MicroRNA dysregulation in colorectal cancer: a clinical perspective

**DOI:** 10.1038/bjc.2011.57

**Published:** 2011-03-01

**Authors:** Y Dong, W K K Wu, C W Wu, J J Y Sung, J Yu, S S M Ng

**Affiliations:** 1 Institute of Digestive Disease and Department of Medicine and Therapeutics, Li Ka Shing Institute of Health Sciences, Prince of Wales Hospital, The Chinese University of Hong Kong, Shatin, NT, Hong Kong, China; 2 Department of Surgery, Prince of Wales Hospital, The Chinese University of Hong Kong, Shatin, NT, Hong Kong, China

**Keywords:** miRNA, colorectal cancer, diagnostic marker, prognostic marker, single-nucleotide polymorphisms

## Abstract

Recent researches have shed light on the biological importance of microRNAs (miRNAs) in colorectal cancer (CRC) genesis, progression and response to treatments. The potential utility of miRNAs in the preclinical stage have been explored and investigated. In this review, we explored the literature and reviewed the cutting edge progress in the discovery of noninvasive plasma and faecal miRNAs for CRC early diagnosis, as well as their measurability and predictability. We also discussed the utility of miRNAs as novel prognostic and predictive markers, and their association with CRC clinical phenotypes including recurrence, metastasis and therapeutic outcomes. Finally, we summarised miRNA-related single-nucleotide polymorphisms and their potential influence on sporadic CRC susceptibility and therapeutic response. In conclusion, the use of miRNAs as biomarker for CRC is still in its infancy and need further characterisation and evaluation.

MicroRNAs (miRNAs), a class of highly conserved ∼22-nucleotides single-stranded RNAs, can act as trans-acting factors that suppress translation or induce messenger RNA (mRNA) degradation of target genes (Bartel, 2004). A spectrum of dysregulated miRNAs was identified to be associated with colorectal cancer (CRC) genesis, progression and therapeutic response. Herein, we summarise recent findings and discuss the potential value of miRNAs as biomarkers for CRC diagnosis, prognosis and susceptibility.

## miRNAs as diagnostic markers

Approximately 1.23 million individuals worldwide will develop CRC and 0.6 million people will die of it annually (Ferlay *et al*, 2010). Fortunately, the mortality and morbidity have modestly declined and CRC has become a preventable disease if pre-neoplastic lesions can be detected with modern screening methods (Lieberman, 2009). Among the available screening approaches, colonoscopy is recommended as the gold standard for high-risk people; however, its invasive nature and expensive cost have hampered the worldwide application. This setting provides the foundation for defining new biomarkers in blood and stool, which can offer more acceptable and practical benefits.

## Plasma miRNAs as diagnostic markers

Surprisingly high concentration of miRNAs is discovered in highly stable, cell-free form in the peripheral blood (Mitchell *et al*, 2008). Circulating miRNAs are packed in complexes, either called exosomes or microvesicles, and released by normal and tumour cells through unknown mechanisms. Emerging evidence has indicated that such external miRNAs are also involved in cell-to-cell signal transduction and genetic information exchange (Kosaka *et al*, 2010). Given that aberrantly expressed miRNAs vary among different tumour types and some of them are secreted into blood (Mitchell *et al*, 2008), circulating miRNAs can potentially serve as noninvasive markers for CRC detection.

We have previously discovered that miR-17-3p and miR-92a (previously named miR-92), both belonging to the miR-17-92 cluster, were significantly elevated in plasma and CRC tissues, but reduced in the postoperative samples when compared with preoperative samples. These data support the idea that CRC cells can release aberrantly expressed miRNAs into the circulatory system. Furthermore, promising discriminatory power of the two miRNAs was verified in an independent larger cohort: at a cut-off value of 3.6 for miR-17-3p (relative expression in comparison with RNU6B), the sensitivity was 64% and the specificity was 70% at a cut-off value of 240 for miR-92a, the sensitivity was 89% and the specificity was 70% (Table 1). In addition, miR-92a could distinguish CRC from other gastrointestinal cancers and inflammatory bowel diseases (Ng *et al*, 2009). Diagnostic potential of miR-92a was further verified by Huang *et al.* They confirmed that miR-92a was significantly increased in CRC plasma compared with healthy control, and the sensitivity and specificity were consistent with our previous study (Table 1). It was also reported that miR-92a could distinguish advanced adenoma from normal control, with a sensitivity of 64.9% and a specificity of 81.4%, whereas its expression levels were not correlated with tumour-node-metastasis (TNM) stages (Ng *et al*, 2009; Huang *et al*, 2010).

Tumour-derived plasma miR-29a is another potential CRC diagnostic marker. It was demonstrated that plasma miR-29a could distinguish CRC and advanced adenoma from normal group with sensitivities of 69 and 62.2%, and specificities of 89.1 and 84.7%, respectively (Table 1). Further studies indicated that the level of miR-29a was significantly increased in CRC compared with adenoma, and that its expression was associated with more advanced TNM stages (Huang *et al*, 2010).

Employing high-throughput transcriptomic sequencing technique, Chen *et al* (2008) found that the serum miRNA expression profiles of CRC and healthy controls were significantly different: 69 miRNAs were only detected in CRC but not in the control group. Nevertheless, it is most likely that only a minor portion of these dysregulated miRNAs can be finally developed into CRC-specific markers, since roughly 80% of the aberrantly expressed miRNAs are also present in the serum of lung cancer patients (Chen *et al*, 2008). It is possible that common carcinogenesis-related miRNAs are shared by different types of tumours or it is merely a tumour-induced ‘systemic reaction’ generated from other sources, such as immune cells. If the latter is the case, future studies should cautiously verify the applicability of a single plasma miRNA, or the use of a combination of multiple plasma miRNAs for specific CRC diagnosis should be recommended.

In previous studies, SYBR green-based real-time polymerase chain reaction (PCR) was generally adopted to detect the presence of miRNA. However, this method has a lower specificity and causes high false-positive and false-negative rates in diagnosis. Recently, Kroh *et al* (2010) described an optimised method of plasma/serum miRNA detection and data analysis. It is expected that the improved protocol can increase the detection accuracy and help to uncover novel miRNA markers in the plasma.

## Faecal miRNAs as diagnostic markers

Stool-based test is widely adopted as noninvasive screening methods for CRC diagnosis. Currently, the most commonly used stool test for CRC screening is the guaiac-based faecal occult blood test (FOBT). However, the test itself has some crucial limitations. Owing to the irregular bleeding nature of colorectal tumours, presence of red meat in digested residues, and other causes of bleeding in the gastrointestinal tract, the specificity and sensitivity of FOBT for CRC are relatively poor. Some advanced adenomas with diameter larger than 1 cm are missed by FOBT (Lieberman, 2009). Other stool-based detections depend on colonocytes that are constantly exfoliated from the intestinal tract as well as from the neoplastic site, which theoretically carry important genetic and epigenetic information for subsequent testing, such as detection of mutation genes or dysregulated mRNAs, proteins or miRNAs.

In contrast with the fast degradation of mRNA and protein, endogenous miRNAs are packed and protected from RNase. They are therefore more likely to be detected. Nonetheless, the stool environment is much more complex and hostile than plasma, and human RNA is expected to constitute <1% of totally stool RNA. In this regard, several criteria, including measurability, reproducibility and predictability, must be met if stool miRNAs have to be developed as diagnostic markers. It is a ground-breaking attempt that Ahmed *et al* (2009a) evaluated the feasibility of using miRNA from faecal specimens as screening markers for CRC. They developed a novel detection protocol including stool preparation, stool miRNA extraction and quantitative analysis. Commercial miRNA extraction kit is also available for stool miRNA purification, which can yield total RNA of high quality and integrity for further assays. Taqman-based quantitative real-time PCR is performed for miRNA quantification, which can eliminate the interference from food and intestinal bacteria. At present, this analysis protocol is most widely adopted and further optimised by other studies. For example, Koga *et al* (2010) used immunomagnetic beads conjugated with EpCAM monoclonal antibody to isolate colonocytes from stool, rather than performing colonocytes scrapping from the stool mucinous layer; several other studies used homogenised stool specimens to reduce selection bias. A simpler method known as direct miRNA analysis, which analysed extracellular miRNA in stool without RNA extraction has also been recently developed (Link *et al*, 2010). It is necessary to compare different methods in future studies and an appropriate balance between precision and costs should be considered for further clinical use.

A key issue of miRNA detection in stool is the selection of endogenous control. Although several small RNA species are recommended for normalising miRNA expression in tissues and cell lines (Wong *et al*, 2007), the widely used RNU6B expression is not correlated with total RNA concentration in stool (Link *et al*, 2010). In addition, RNU6B is rapidly degraded and the detection rate was relatively low in stool specimens. As there is no consensus on suitable control for stool testing, it is suggested that quantification should be performed with equal amount of starting total RNA or adopting other stably expressed small RNAs as normaliser, such as the classical 18S rRNA control and miR-16.

It has been reported that the expression levels of several miRNAs can distinguish healthy control and ulcerative colitis from CRC, as well as to differentiate among CRC with different Dukes' stages. miR-21, miR-106a, miR-96, miR-203, miR-20a, miR-326 and miR-92 showed a higher expression level in stool of CRC patients with advanced Dukes' stages, while miR-320, miR-126, miR-484-5p, miR-143, miR-145, miR-16 and miR-125b exhibited lower expression in stool of CRC patients with advanced Dukes' stages. It is noteworthy that Ahmed *et al* (2009a) cannot provide a sufficient statistical power for each miRNA because of small sample size used in the study. Moreover, their conclusion was challenged by other studies. Link *et al* (2010) reported that the levels of stool miR-21 and miR-106a were significantly increased in patients with CRC and adenoma compared with normal controls, but were negatively associated with tumour stages. On the other hand, the expression levels of miR-143 as well as miR-17, miR-622 and miR-654-3p were found to have no significant difference between colorectal neoplasia and normal control.

On the basis of screening of 59 patients and 74 normal controls, we previously reported that stool miR-92a and miR-21 levels were significantly higher in CRC compared with normal controls. At a cut-off value of 430 copies per ng of extracted stool RNA, miR-92a had a sensitivity of 50% and a specificity of 80% towards CRC. At a cut-off value of 820 000 copies per ng, miR-21 had a sensitivity of 50% and a specificity of 83% (Table 1). However, stool miR-92a cannot distinguish adenoma and CRC, or different CRC stages (Wu *et al*, 2010). In the largest cohort to date, Koga *et al* (2010) reported the stool miR-17-92 cluster and miR-135 significantly increased in CRC patients, with sensitivities of 69.5 and 46.2%, and specificities of 81.5 and 95.0%, respectively (Table 1). In addition, the sensitivities of the stool miR-17-92 cluster and miR-135 were lower for the proximal region tumour detection. Although the expression level of miR-21 increased in cancer tissue, stool miR-21 showed no difference between CRC patients and healthy volunteers.

It has been postulated that tumour cells and most tumour markers can be readily detected in stool at earlier stages of CRC than in blood. Thus, stool miRNA testing has the advantage for pre-cancerous lesion screening (Ahlquist, 2010). On the other hand, colonocytes shed from proximal region will transit a longer distance and will be more exposed to cytolytic agents, thus less likely to be preserved and sampled. If this is a common phenomenon for stool miRNA markers, right-sided CRC will have less chance to be detected by this approach. Although the use of stool miRNAs as biomarkers is still in its infancy and none of the miRNA discussed above has progressed beyond the preclinical stage, the available data indicated that higher specificity, sensitivity and reproducibility can be achieved for stool miRNA detection. Further studies of stool miRNA characterisation and validation are needed for CRC diagnosis.

## miRNAs as prognostic markers for CRC

In spite of the improvement in surgical technique, CRC patients who have an operable tumour will still have a high risk of subsequent locoregional relapse. On the other hand, the clinical responses to adjuvant therapy vary among individuals. Recently, the utility of miRNA as prognostic markers for CRC has been extensively studied.

Several preclinical and clinical studies have highlighted the potential of miR-21 as a highly promising prognostic marker. Slaby *et al* (2007) reported that miR-21 upregulation in CRC patients were associated with lymph node positivity and development of distant metastasis based on their study of 29 patients. Later, a larger scale study using miRNA array was conducted with two independent cohorts with different races and geographical distributions. Schetter *et al* (2008) identified 37 aberrantly expressed miRNA in CRC, among which 5 highly expressed miRNA (miR-20a, miR-21, miR-106a, miR-181b and miR-203) were associated with poor survival in the test cohort. Further validation confirmed the statistically significant association between poor prognosis and high tumour level of miR-21 in Asian CRC patients. In addition, high miR-21 expression level was found to be associated with poor response to adjuvant therapy and more rapid recurrence in patients with stage III disease. Kulda *et al* (2010) confirmed that higher miR-21 level in primary tumour was correlated with shorter disease-free interval (DFI), but not with overall survival (OS). Although significantly higher level of miR-21 was also found in liver metastasis of CRC, its level was not associated with DFI or OS. miR-31 is another crucial factor for tumour metastasis and it is dysregulated in both CRC cell lines and tissues. Bandrés *et al* (2006) found that the expression level of miR-31 was significantly increased in stage IV compared with stage II CRC. Wang *et al* (2009) confirmed the association between miR-31 level and pathological stages, including local invasion. Numerous other miRNAs (Table 2) are also shown to have potential predictive value for prognosis, but further large-scale studies are needed for their validation.

It is known that microsatellite status is associated with CRC disease course and response to adjuvant therapy. Lanza *et al* (2007) first suggested a link between microsatellite status and different miRNA profiles, and identified 14 differentially expressed miRNA between microsatellite stable tumour (MSS) and microsatellite unstable tumour-high (MSI-H). Employing miRNA array on 49 specimens from TNM stage II CRC patients, Schepeler *et al* (2008) showed a signature consisting of 4 miRNAs (miR-142-3p, miR-212, miR-151 and miR144) could significantly separate MSI from MSS tumours, with 92% sensitivity and 81% specificity. Furthermore, they reported that 17 miRNAs could separate the MSS subtype according to the metastatic recurrence status, resulting in 81% accuracy, 77% sensitivity and 83% specificity. Among the 17 miRNAs, high levels of miR-320 and miR-498 were associated with longer progression-free survival. Moreover, subgroups of MSI (MSI-high (MSI-H) and MSI-low (MSI-L)) could be distinguished with miRNAs. Earle *et al* (2010) later reported that upregulation of miR-92, let-7a and miR-145 were associated with MSI-L status whereas increased level of miR-155, miR-223, miR-31 and miR-26b were correlated with MSI-H status.

## miRNAs as predictive markers for treatment outcome in CRC

Fluoropyrimidine-based adjuvant therapy is commonly used in CRC treatment. Patients who respond to S-1, the fourth-generation Fluoropyrimidine, showed lower levels of miR-181b and let-7g. However, neither miR-181b nor let-7g was associated with survival (Nakajima *et al*, 2006). Moreover, by induction G_2_ phase arrest through downregulation of denticleless protein homolog in a p53-dependent manner, miR-215 increased chemoresistance of HCT116 to methotrexate and tomudex, but had no impact on cisplatin and doxorubicin (Song *et al*, 2010). Remarkably, although miR-215 was downregulated in clinical colon cancer specimen, high level of miR-215 was found in colon cancer stem cells. Cetuximab, the monoclonal antibody that inhibits epidermal growth factor receptor signal transduction, is used to treat metastatic or advanced CRC with wild-type KRAS. Ragusa *et al* (2010) found that miR-146b-3p and miR-486-5p were more abundant in mutant KRAS patients compared with wild-type ones. Moreover, they suggested that the downregulation of let-7b and let-7e and upregulation of miR-17-3p were potential predictive markers of cetuximab resistance.

Several miRNAs are also implicated in the modulation of radiosensitivity. Svoboda *et al* (2008) reported that median levels of miR-125b and miR-137 were upregulated in rectal cancer patients after a short-course of capecitabine-based chemoradiotherapy, and higher induction of miR-125b and miR-137 were associated with worse response to the treatment. Moreover, Ahmed *et al* (2009b) found different miRNA expression patterns of colon cancer cell line HT29 treated with two clinical X-irradiation modalities. Nonetheless, their clinical values remain unexplored.

## miRNA-related single-nucleotide polymorphisms influenced risk of sporadic CRC and therapy response

The whole genome is constantly evolving and generates many germ-line nucleotide changes known as single-nucleotide polymorphisms (SNPs). SNPs that fall into the protein coding sequence may have a biological effect by changing the amino acid sequence or yielding a premature stop code. Although SNPs residing in the untranslated region (UTR) do not alter the function of the protein, they may occasionally perturb the protein expression level and may have pathogenic consequences (Yu *et al*, 2007). As a result of the stringent sequence requirement of miRNA, it is speculated that SNPs residing in miRNA-associated motifs in the 3′ UTR, such as the seed region and position 13–16, will potentially interfere with the formation of miRNA::mRNA complex. It raises the possibility that individuals with altered miRNA target site within tumour-related genes are more prone to develop CRC. The initial study of association between SNPs at miRNA-binding sites and CRC risk was reported by Landi *et al* (2008). The selected 104 candidates are well-known genes that are related to inflammation, prostaglandins and thromboxane synthesis, obesity and insulin resistance. By *in silicon* searching for the SNPs in 3′ UTR and assessing the variation of Gibbs energy between the alleles and validation in a case–control study, they reported that both heterozygotes and homozygotes of SNP (rs17281995) within CD86 showed a statistically significant association with increased risk of CRC in Caucasians (especially the risk of rectal cancer), and that the G>C substitution would weaken the affinity of miR-337, miR-582 and miR-200a^*^ (Landi *et al*, 2008). Another SNP that less strongly associated with CRC risk is rs1051690 within insulin receptor (INSR), which would theoretically impact miR-612 and miR-618 (Landi *et al*, 2008) (Table 3). One more thing that should be addressed is the predicted miRNAs may possibly be false-positive errors, because the algorithm is updating and all the five miRNAs predicted in Landi's paper are not found in the latest miRNA online databases. Thus, whether illegitimate miRNA target site can cause higher CRC risk needs to be experimentally verified in the future.

Ultraconserved region (UCR) encodes a class of non-coding RNAs and their expression can be regulated by miRNA *in vivo* (Calin *et al*, 2007). Previous studies indicated that SNPs at UCR loci are related to cancer susceptibility (Yang *et al*, 2008). Wojcik *et al* (2010) randomly selected and sequenced 28 UCRs in Caucasians and identified 4 UCRs harboured sequence variations in 35 CRC patients, but none of them existed in a cohort of 175 cancer-free control. The A>G substitution at position 90 of *uc*.276 in CRC patients would putatively disturb the interaction with miR-214 and miR-887.

Single-nucleotide polymorphisms located within the miRNA biogenesis machinery or the miRNA itself may also have possible influences on clinical outcomes. Exportin 5 (XPO5) is responsible for the miRNA::miRNA duplex cytoplasm translocation. Inactivated XPO5 trapped many precursor miRNA that had potential tumour-suppressor feature in the nucleus, and promoted cancer growth *in vitro* and *in vivo* (Melo *et al*, 2010). Boni *et al* (2010) discovered a trend of association between SNP (rs11077) in 3′UTR of XPO5 and disease control rate. It is not clear whether this SNP influence protein level of XPO5 as well as precursor miRNA shuttling. Lee *et al* (2010) reported that 23 SNPs (including rs11077) in the functional regions of miRNA biogenesis machinery did not influence survival of CRC patients. In addition, SNP (rs1834306) in 5′UTR of pri-miR-100 was weakly associated with CRC progression while C>T substitution (rs7372209) in 5′UTR of pri-miR-26a-1 was significantly associated with disease progression and therapy response (Boni *et al*, 2010). However, 17 SNP located in miRNA precursors, with inclusion of rs1834306 and rs7372209, did not influence the OS rate in a study of 426 surgically treated Korea CRC patients (Lee *et al*, 2010).

Single-nucleotide polymorphisms and their mechanisms related to CRC progression remain to be investigated. Since SNPs in the miRNA-binding motifs are sometimes deleterious, natural selection keeps the SNP density in conserved seed region at lower level than expected, approximately 0.50 SNP per kb (Chen and Rajewsky, 2006). Nevertheless, genome-wide screening for miRNA-related SNPs and understanding of their biological relevance can make further advances in the evaluation of individual CRC susceptibility, expending the list of CRC-associated genes, and ultimately help to develop patient-tailored medicine.

## Future perspective

Genome-wide searching can help identify many oncogenic and tumour-suppressing miRNAs, which are potential therapeutic targets for CRC treatment. Theoretically, the downregulated miRNAs can be replaced by chemically synthesised mimics. Overexpressed miRNAs can also be targeted by anti-miRNA oligonucleotides. Recently, Idogawa *et al* (2009) reported that adenovirus mediated cocistronic expression of the p53 protein and artificial miRNA that targeted p21 increased the chemosensitivity of infected CRC cells. However, without fully understanding their biological functions, manipulation of miRNA can potentially induce severe side-effects as miRNA will influence a spectrum of genes. In addition, the technique of target-specific delivery of miRNA is immature. Many issues must be addressed before the preclinical findings can be translated into clinical benefits.

In conclusion, those studies mentioned above highlight the potential value of miRNAs in CRC diagnosis, prognosis and susceptibility. Further large-scale evaluation in multiple independent cohorts is indispensable for determining their realistic expectation.

## Figures and Tables

**Table 1:** miRNAs as potential diagnostic markers for CRC

**miRNA**	**Sensitivity, %**	**Specificity, %**	**AUC**	**Sample size, *n***	**Endogenous control**	**Remarks**	**Reference**
*Plasma miRNA*							
miR-17-3p	64	70	CRC: 0.717 (95% CI: 0.63–0.80)	140 (90 CRC, 50 control)	RNU6B	Upregulated in CRC plasma	Ng *et al* (2009)
miR-92a	89	70	CRC: 0.885 (95% CI: 0.83–0.94)	140 (90 CRC, 50 control)	RNU6B	Upregulated in CRC plasma, could distinguish CRC from other GI cancer and IBD, not associated with TNM stages	Ng *et al* (2009)
miR-92a	84	71.2	CRC: 0.838 (95% CI: 0.775–0.900)	196 (100 CRC, 37 advanced adenomas, 59 control)	miR-16	Upregulated in CRC plasma, not associated with TNM stages	Huang *et al* (2010)
	64.9	81.4	Advanced adenomas: 0.749 (95% CI: 0.642–0.856)				
miR-29a	69	89.1	CRC: 0.844 (95% CI: 0.786–0.903)	196 (100 CRC, 37 advanced adenomas, 59 control)	miR-16	Upregulated in CRC plasma, associated with advanced TNM stages	Huang *et al* (2010)
	62.2	84.7	Advanced adenomas: 0.769 (95% CI: 0.669–0.869)				
							
*Faecal miRNA*							
miR-92a	50	80	—	133 (59 CRC, 74 control)	Equal amount of total RNA	Upregulated in stool of CRC patients	Wu *et al* (2010)
miR-21	50	83	—	133 (59 CRC, 74 control)	Equal amount of total RNA	Upregulated in stool of CRC patients	Wu *et al* (2010)
miR-17-92 cluster	69.5	81.5	—	316 (197 CRC, 119 control)	U6 snRNA	Upregulated in stool of CRC patients	Koga *et al* (2010)
miR-135	46.2	95	—	316 (197 CRC, 119 control)	U6 snRNA	Upregulated in stool of CRC patients	Koga *et al* (2010)

Abbreviations: AUC=area under the ROC curve; CI=confidence interval; CRC=colorectal cancer; GI cancer=gastrointestinal cancer; IBD=inflammatory bowel disease; miRNA=microRNA; snRNA=small nuclear RNA; TNM=tumour-node-metastasis.

**Table 2:** miRNAs as potential prognostic and predictive markers for CRC

**miRNA**	**Dysregulation^a^**	**Clinical-related phenotypes**	**Reference**
let-7g	Upregulated in CRC	Higher level associated with poor S-1 response	Nakajima *et al*, 2006
miR-18a	Upregulated in CRC	Higher level associated with poor overall survival	Motoyama *et al*, 2009
miR-21	Upregulated in adenoma, CRC, and liver metastasis tissue	Higher level associated with lymph node positivity, metastasis; poor survival, poor therapeutic outcomes, rapid recurrence; shorter disease-free interval	Slaby *et al*, 2007; Schetter *et al*, 2008; Kulda *et al*, 2010
miR-31	Upregulated in CRC	Higher level associated with higher TNM stages and local invasion	Bandrés *et al*, 2006; Slaby *et al*, 2007; Wang *et al*, 2009
miR-106a	Upregulated in colon cancer	Higher level associated with longer disease-free survival and overall survival	Díaz *et al*, 2008; Schetter *et al*, 2008
miR-143	Downregulated in colon cancer and liver metastasis tissue	Lower level associated with larger tumour size and longer disease-free interval	Slaby *et al*, 2007; Motoyama *et al*, 2009; Wang *et al*, 2009; Kulda *et al*, 2010;
miR-145	Downregulated in CRC	Lower level associated with large tumour size; related with tumour location	Slaby *et al*, 2007; Motoyama *et al*, 2009 Wang *et al*, 2009;
miR-181b	Upregulated in CRC	Higher level associated with poor S-1 response	Nakajima *et al*, 2006
miR-320	Downregulated in MSS tumour	Lower level associated with shorter progression-free survival	Schepeler *et al*, 2008
miR-498	Downregulated in MSS tumour	Lower level associated with shorter progression-free survival	Schepeler *et al*, 2008

Abbreviations: CRC=colorectal cancer; miRNA=microRNA; MSS=microsatellite stable tumour; TNM=tumour-node-metastasis.

a Up- or downregulation is stated as miRNA expression relative to normal colon tissue, or MSS relative to MSI.

**Table 3:** Nucleotide polymorphisms in CRC patients and potentially related miRNAs

**db SNP ID**	**Location**	**Variation (M/m)**	**Potentially related miRNA**	**Remark**	**Reference**
rs17281995	3′UTR of CD86	G/C	miR-337 miR-582 miR-200a^*^	Associated with increased risk of CRC in Caucasians	Landi *et al* (2008)
rs1051690	3′UTR of INSR	G/A	miR-612 miR-618	Associated with increased risk of CRC in Caucasians	Landi *et al* (2008)
—	Position 90 in *uc*.276	A/G	miR-214 miR-887	Rare polymorphism found in CRC patients	Wojcik *et al* (2010)
rs11077	3′UTR of XPO5	A/C	—	Weak associated with DCR; not associated with survival	Boni *et al* (2010); Lee *et al* (2010)
rs7372209	5′UTR of pri-miR-26a-1	C/T	miR-26a-1	Significantly associated with ORR and TTP; not associated with survival	Boni *et al* (2010); Lee *et al* (2010)
rs1834306	5′UTR of pri-miR-100	C/T	miR-100	Weak associated with TTP; not associated with survival	Boni *et al* (2010); Lee *et al* (2010)

Abbreviations: CRC=colorectal cancer; DCR=disease control rate; ORR=overall response rate; M=major allele; m=minor allele; miRNA=microRNA; SNP=single-nucleotide polymorphism; TTP=time to progression; UTR=untranslated region.
